# Identification of metabolic system parameters using global optimization methods

**DOI:** 10.1186/1742-4682-3-4

**Published:** 2006-01-27

**Authors:** Pradeep K Polisetty, Eberhard O Voit, Edward P Gatzke

**Affiliations:** 1 Department of Chemical Engineering, University of South Carolina, Swearingen Engineering Center, 301 Main Street, Columbia, SC 29208, USA; 2 The Wallace H. Coulter Department of Biomedical Engineering, Georgia Institute of Technology and Emory University, 313 Ferst Drive, Suite 4103, Atlanta, GA 30332, USA

## Abstract

**Background:**

The problem of estimating the parameters of dynamic models of complex biological systems from time series data is becoming increasingly important.

**Methods and results:**

Particular consideration is given to metabolic systems that are formulated as Generalized Mass Action (GMA) models. The estimation problem is posed as a global optimization task, for which novel techniques can be applied to determine the best set of parameter values given the measured responses of the biological system. The challenge is that this task is nonconvex. Nonetheless, deterministic optimization techniques can be used to find a global solution that best reconciles the model parameters and measurements. Specifically, the paper employs branch-and-bound principles to identify the best set of model parameters from observed time course data and illustrates this method with an existing model of the fermentation pathway in *Saccharomyces cerevisiae*. This is a relatively simple yet representative system with five dependent states and a total of 19 unknown parameters of which the values are to be determined.

**Conclusion:**

The efficacy of the branch-and-reduce algorithm is illustrated by the *S. cerevisiae *example. The method described in this paper is likely to be widely applicable in the dynamic modeling of metabolic networks.

## Background

The past few years have witnessed an enormous increase in the availability and quality of high-throughput data characterizing the status of cells at the genomic, proteomic, metabolic and physiological levels. In most cases, these data were interpreted as simple snapshots or in a comparative setting with the goal of differentiating between normal and perturbed or diseased cells. It is now becoming feasible to use the same methods to record the status of cells over time. The resulting time series data contain enormous amounts of information on the dynamics of functioning cells. Several groups of scientists around the world have begun to develop methods for inferring from these profiles the underlying functional networks at the genomic or metabolic level. In principle, this task is a straightforward matter of defining a suitable model and estimating its structure, but numerous conceptual and computational difficulties have made the implementation of this inverse problem challenging. The difficulties fall into several categories.

First, it is necessary to select a mathematical modeling framework that is rich enough to capture the observed dynamics with sufficient accuracy but that is also structured in a fashion that allows interpretation of results beyond pure parameter estimation. For instance, if one selected a high-order polynomial and estimated parameters capturing the observed time profiles, then the resulting coefficients would not have much meaning and could hardly be translated into biological insight. Several groups [1-10] have therefore focused on S-system models within the modeling framework of Biochemical Systems Theory (BST), which itself has a rich history of successful analysis and application. S-systems, and the alternative variant of Generalized Mass Action (GMA) systems, have the advantage for inverse tasks that their parameter values map essentially one-to-one onto structural and regulatory features of biological networks. Thus, structure inference is reduced to the simpler task of parameter estimation. BST has been the subject of several hundred articles, reviews, books, chapters, and presentations [11-21], which permits us to review only a few features that are of particular interest here.

The second difficulty arising in the inference problem is the preparation of data and the preprocessing of the task itself. Clearly, noise in the data complicates the estimation and often leads to local minima in the search space, as well as to unwanted redundancies in inference. Furthermore, the fact that essentially any model of a dynamic biological system involves differential equations necessitates efficient integrators, because these methods may consume in excess of 95% of the time required to estimate parameters in systems of differential equations [9]. In order to reduce this computational cost, several groups have devised methods addressing some of these problems. One efficient strategy is the estimation of slopes of the profiles, which permits the replacement of differentials with the estimated slopes at many time points and consequently the conversion of systems of differential equations into larger systems of more easily computed algebraic equations [3,9,18,22,23]. A prerequisite of this method is obviously the reliable estimation of slopes, for which various smoothing methods have been proposed, including neural network smoothing [3,9], filtering [6] and collocation methods [8]. It was also shown that decoupling of systems of *n *differential equations may be achieved by treating *n*-1 of the data sets as inputs in the remaining equation [2]. In complementary approaches, the search process was simplified by making use of auxiliary information about the biological system, which was translated into constraints on the parameters that had to be estimated [3], and by priming the search process with reasonable initial guesses, which were obtained directly from the topology of the systems [24] or through various means of linearization [25].

The third difficulty of the inverse problem is the estimation of parameters itself. Because of the inherent nonlinearities, the task is hampered by trapping of the search algorithm in local minima, lack of convergence, or a convergence speed that makes inference of larger systems infeasible. In the past, this step has probably received the least attention, and the various groups addressing the inverse problem have resorted to standard methods of nonlinear regression, genetic algorithms or simulated annealing. In this article, we address this subtask of the inference problem by replacing the regression step with a global optimization algorithm. Specifically, we formulate the nonconvex estimation problem as a global optimization task that uses branch-and-bound principles to identify the best set of model parameters given observed time profiles. The greatest advantage of this method is that it guarantees that the optimum obtained is global within pre-defined bounds on the parameter search space. Note that global optimization does not guarantee that the resulting solution is unique; the method guarantees that no other points exist with a better objective function than the global solution. Multiple degenerate solution points could exist with identical unique objective function values. As an example, we estimate the parameters of a model describing the fermentation pathway in *Saccharomyces cerevisiae*, as described in [26]. This system has five dependent states and a total of 19 unknown parameters. It is manageable in size, yet representative of the nonlinearities typically encountered in metabolic modeling and has therefore been used for a variety of analyses in the past [18,27-29].

### Model formulation

Metabolic pathway analysis is concerned with the modeling, manipulation and optimization of biochemical systems. While valuable insights may be gained from the formulation of these systems as stoichiometric networks [30], whose functioning may be constrained as described in Flux Balance Analysis [31], it will ultimately be necessary for many purposes to formulate the processes as dynamical systems that account for detailed kinetic features, such as the regulation and modulation of enzyme-catalyzed steps and transport processes. The default approach for this purpose may seem to be a model representation in the tradition of Michaelis and Menten. However, it was recognized early on that this representation is not particularly well suited for the analysis of large networks [32-34], and this led to the development of alternative methods, among which BST, Metabolic Control Analysis [35] and the "log-linear" approach [36] have received the most attention. In particular, it was shown that the S-system variant within BST has favorable features for the optimization of nonlinear metabolic systems [15,37]. As an alternative to the S-system formulation, which may be criticized for its manner of flux aggregation, the GMA variant overcomes this issue, even though it loses some of the advantages of the S-system form such as its linearity at steady state [12] and its sometimes slightly higher accuracy [38,39]. GMA representations have the advantage that they are closer to biochemical intuition and that production and degradation terms do not vanish if one of the contributing fluxes disappears, as is the case with S-systems. GMA systems are also interesting in that they include both stoichiometric systems and S-systems as special cases so that they allow a seamless transition from linear to fully kinetic models. Our task in this article is thus the estimation of GMA parameters from time series.

In the GMA formulation within BST, the change in each dependent pool (state variable) is described as a difference between the sums of all fluxes entering the pool and all fluxes leaving the pool. Each flux is individually linearized in logarithmic coordinates, which in Cartesian coordinates corresponds to a product of power-law functions that contains those and only those variables that directly affect the flux, raised to an exponent called its kinetic order. The product also contains a rate constant that determines the magnitude of the flux or speed of the process. The mathematical formulation of any GMA model is thus



where *γ*_*i*1_,...,*γ*_*ik *_are rate constants corresponding to *k *reactions of production/consumption, and *ζ*_*ijk *_are kinetic orders for species *i *in reaction *k *involving species *j*. In cases where species *j *does not have any influence on a given power-law term, *ζ*_*ijk *_= 0. The number of reactions in one differential equation, *k*, may be different for each species. Reaction terms for consumption of one species may appear as a production term for another species. The system consists of *n *differential equations, representing the time-dependent variables, but also contains *m *time-independent variables that affect the system but are not affected by the system and are typically constant from one experiment to the next. The power-law terms in Eq. (1) are the result of a straightforward Taylor approximation, which is applicable to an essentially unlimited variety of underlying processes and may include different types of interactions, activation, inhibition and processes associated with dilution and growth.

It is interesting to note that every parameter of a GMA model has its unique role and interpretation. This situation is significantly different from using unstructured fitting models such as higher-order polynomials or splines. In a generic polynomial representation, every coefficient is likely to change if additional data points are used for fitting or if points are removed. Thus, except for the fact that the higher-order coefficients are associated with higher derivatives, which otherwise are not very meaningful, not much can be said about their biological role in the modeled process. In the GMA model, by contrast, every parameter has a unique meaning in the subject area of the model. Each kinetic order quantifies solely the effect that a particular variable has on a given process. For example, the first kinetic order in a later example is *ζ*_121 _= -0.2344 (see Figure 7). Thus, it uniquely describes the effect of metabolite *X*_2 _on the first production process of *X*_1_. The effect is inhibitory, which is indicated by the negative sign, and is only moderately strong, which is reflected in the small magnitude of the parameter. In this fashion, there is a one-to-one relationship between kinetic orders and structural features of the model. The interpretability of the parameters can also been seen from a different point of view: in principle, each kinetic order can be obtained directly from local information on the system. Namely, if it possible to vary *X *_*j *_while keeping all other variables constant, and to measure the consequent changes in the production of *X*_*j*_, then the slope of the production process as a function of *X*_*j*_, in log space, is exactly the kinetic order in question. This type of interpretation is usually not possible in global fitting models such as high-order polynomials. The constant multipliers are rate constants that, as in elemental chemical kinetics, quantify the turnover rate in each process and are always non-negative. Their magnitudes depend on the scales (time, concentration, etc.) of the modeled system.

The S-system representation within BST is formally a special case of a GMA system with at most one positive and one negative term. The correspondence between the biological network and the mathematical representation is slightly different in an S-system because all fluxes entering a pool are collectively linearized in logarithmic coordinates and the same is done with all fluxes leaving the pool. Thus, each S-system equation has at most one influx and one efflux term and thus reads



where *α*_*i*_, *β*_*i *_are the rate constants and *g*_*ij*_, *h*_*ij *_are kinetic orders. These S-system parameters are directly tied to the corresponding GMA system through constraints [18].

Both forms are nonlinear and rich enough to capture any dynamic behavior that can be represented by any set of ordinary differential equations [38]. If set up as alternative descriptions of the same biological system, the two are equivalent at one operating point of choice and typically differ if the system deviates from this point, though the differences are often small in realistic situations [18].

Working with metabolic models consists of three phases: Model design, model analysis and model application. The present work focuses on the first phase of model design. This step is usually executed by assembling a topological map of the phenomenon of interest based on biological knowledge. Kinetic orders and rate constants are then estimated from measured or published kinetic information. In the case of S-systems, it would also be feasible to use steady-state data from multiple experiments with different values of independent variables. Because the steady-state equations of S-systems are linear (in logarithmic coordinates), such data would allow use of a simple matrix inversion leading to optimal parameters, a pseudo-inverse method or a linear programming approach [37]. In the alternative GMA approach, logarithmic transformation does not completely transform the estimation problem into a linear formulation, thus requiring nonlinear methods.

In addition to this traditional bottom-up approach, a top-down approach is becoming increasingly feasible. This complementary approach is based on time profiles that are used for the determination of system parameters through some type of estimation and their interpretation in terms of structural and regulatory information. We describe in the following how this estimation is facilitated with a branch-and-bound method, which has not previously been used for this purpose.

### Optimization formulation

The goal of model identification is to estimate the "best" set of parameter values, which minimizes the error between the process data and the model response. This parameter estimation problem can be formulated as a nonconvex nonlinear optimization problem and can therefore be solved using global optimization techniques. The parameters to be estimated in GMA systems are rate constants *γ *and kinetic orders *ζ *as shown in Equation 1 leading to the following formulation:



The parameter *r *denotes the r-norm considered for minimization. It usually takes the values 1 (min sum absolute), 2 (min sum square), or ∞ (min max error). *P *is the number of data points sampled at times *t*, *h*_*i *_are the nonlinear rate expressions from Eq. (1) that define the production and consumption rates for species *i *given vectors of model parameters *γ *and *ζ*, *e*_*i*_(*t*) are the errors associated with each constraint equation for species *i *at time *t*, and *n *is the number of dependent variables. In the formulation described above, the objective function is linear because the maximum absolute error is minimized. The nonconvexity arises from the equality constraints, which are nonlinear. It is useful to split these nonlinear equality constraints into two inequality constraints, at least one of which will be nonconvex.

Following strategies proposed in the literature [6,8,9], it is possible to smooth the raw time profiles, which subsequently allows the computation of slopes at many data points and thus the replacement of differentials on the left-hand side of Equation 1 with estimated slopes. Thus, assuming that the rate of change of each species is obtainable at each desired time point, (*t*), and the values of the concentrations *X*_*i*_(*t*) at time *i *are known, the optimization task in Eq. (3) can be formulated as a general nonconvex nonlinear programming problem in the form shown in Eq. (4):



where the vector *x *∈ **R**^*N *^is a vector of *N *unknowns including the error terms and unknown parameters and *f*(*x*), *g*_*k*_(*x*) : *R*^*N *^→ *R*^1^. Here, the index *k *represents constraint *k *of *m *total constraints.

This formulation is rather general in that the functions *f*(*x*) and *g*_*k*_(*x*) may be nonlinear and nonconvex. In particular, the formulation allows the estimation of globally optimal GMA systems, given time profiles. Deterministic methods for global optimizations of this type depend on the generation of convex function relaxations of the nonconvex nonlinear functions. Numerous methods have been proposed for constructing such relaxations. For this work, we use a reformulation method for factorable expressions [40]. This method converts the original factorable nonconvex nonlinear problem into an equivalent form through the introduction of new variables *z*_*ik *_for every product of power-law terms at measurement sample time *t *in the system [15]:



Note that the species concentration *X*_*i*_(*t*) in the preceding equation is assumed to be known from the observed data. If it is not, it may be obtained through interpolation from a preparatory smoothing of the observed data [3,9]. If no information at all is available on the variable, the GMA representation should probably be reduced in complexity [18]. As an example for such a reduction, suppose that *X*_1 _is converted into *X*_2 _and *X*_2 _is converted into *X*_3_. If *X*_2 _is not observable, then one would probably formulate the system without *X*_2_, and make *X*_3 _a function of *X*_1_. Since the mathematics underlying the GMA representation is directly based on Taylor's theorem, *X*_3 _then simply becomes a power-law term containing *X*_1_. Such omissions of variables have been discussed in the literature [31,41].

For simplicity of discussion, we assume that a complete data set is available. The problem specified in Eq. (3) is then of the form:



where *s*_*ik *_for reaction *k *of production or consumption of species *i *is either +1 or -1. The variables in this reformulated problem can be collected into the vectors *z*, *e*, *γ*, and *ζ*

To obtain efficacious solutions, the problem must be formulated such that it contains only linear and "simple" nonlinear constraint functions for which one can construct relaxations that use the convex envelopes already known for simple algebraic functions. The nonlinear equality constraints in the original GMA formulation become simple (linear) sums when they are expressed in the new variables, *z*. Furthermore, taking the logarithm of each definition for *z *from Eq. (5) leads to a new set of linear equations and simple nonlinear equations. The connection between these two sets is a set of simple logarithmic constraints. To streamline notation, the additional new variables are defined as: *w*_*ik *_= *ln*(*z*_*ik*_) and Γ_*ik *_= *ln*(*γ*_*ik*_). Thus, we obtain a sum of logarithmic functions for each term *z*_*ik*_(*t*), namely:



For the purpose of convexification, one can omit the logarithm of the rate constant because it simply shifts the optimal solution by a constant. This results in the following formulation:



As *s*_*ij*_, *X*_*i*_(*t*), and (*t*) are known at time values *t*, the only unknowns are *w *Γ, *z*, *ζ *and *e*. Values for *γ *can be determined easily when the solution value for Γ is found. All constraints other than *w*_*ik*_(*t*) = *ln*(*z*_*ik*_(*t*)) are linear.

As mentioned earlier, the parameter *r *in Eq (3) denotes the r-norm considered for minimization. In the case with *r *= 1, the sum absolute value of the error, which is linear, is desired for minimization. In the case of *r *= 2, the objective function is nonlinear, but at least convex. This nonlinearity increases the complexity of the task during the generation of problem relaxations, requiring additional variables and constraints in the linear relaxation. It is well known that when minimizing the absolute value of some variable, constraints of the form |*f*(*x*)| = *e *can be written as two inequality constraints, *f*(*x*) ≤ *e*, and -*f*(*x*) ≤ *e*. The formulation then may be written as follows:



Note that the variables Γ_*ik*_, *ζ*_*ijk *_and *e*_*i*_(*t*) can be consolidated into the vector *y*. The variables in this vector *y *only appear in linear constraints, while *w *and *z *are related through a simple nonlinear expression. Thus, in comparison with the formulation of Equation 4, the variables *x *include *w*, *z *and *y*. The objective function *f*(*x*) and many of the constraint functions *g*_*k*_(*x*) are linear. The objective function (1) represents a sum of the absolute value of errors on the balance equations. The balance equations in (2) and (3) relate the rate of change, (*t*) of the species at time *t*, to the individual reaction rates that consume or produce that species, *z*_*ik*_(*t*), as well as the absolute error for that equation at that point in time. Constraints (4) and (5) result from the transformation of the power-law expressions for the rate equations.

**Figure 1:**
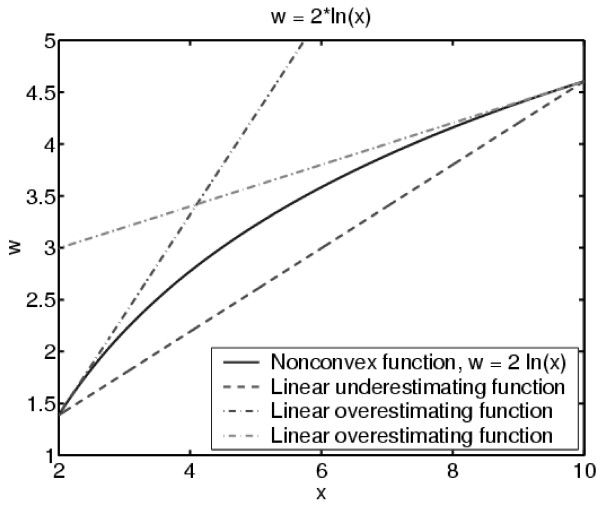
Convex relaxation using linear constraints.

The convex relaxation using linear constraints for a logarithmic function is illustrated in Figure 1. In this figure, the solid line corresponds to the nonlinear function *w *= 2 * *ln*(*x*), the dashed line represents the linear underestimating function, and the dash-dot lines serve as the linear overestimating functions.

As bounds are known for the concave nonconvex function, the secant can be used as a linear underestimation function. Multiple outer approximation linearizations can be used as linear overestimation functions. The intersection of these linear constraints is a relaxation of the original nonlinear function.

Taking the formulation in Eq. (9), coefficients for the linear inequality constraints are the constant values of (*t*) and *s*_*ik*_. The linear equality constraint coefficients include the constant values *ln*(*X*_*j*_(*t*)), while *e*, *z*, *w*, Γ and *ζ*, are the unknowns. The reformulated nonconvex Non-Linear Programming (NLP) problem is now in the form



where all linear inequality constraints are represented by *A*_1_[*w*^*T *^*z*^*T *^*y*^*T*^]^*T *^≤ *b*_1_, and *A*_2_[*w*^*T *^*z*^*T *^*y*^*T*^]^*T *^= *b*_2 _defines the new linear constraints obtained from reformulation, while *w *= *η*(*z*) provides the relationship between *w *and *z*. Bounds on *w *are determined from the bounds on *z *using interval methods. Note that *η *consists of simple nonlinear (logarithmic) terms and that the formulation in Equation 10 is the same as that in Equation 4 with the vector of unknowns *x *made up of *w*, *z *and *y*. Additionally, *f*(*x*) and many of the *g*_*k*_(*x*) are linear functions in Eq. (4). Note that any equality constraint *g*_*k*_(*x*) = 0 can be written equivalently with two inequalities as 0 ≤ *g*_*k*_(*x*) ≤ 0 or *g*_*k*_(*x*) ≤ 0, -*g*_*k*_(*x*) ≤ 0.

Convex relaxations for this task are constructed with DAEPACK [42,43], an automated code generation tool. The advantage of using the DAEPACK tool for this purpose is that it can be applied directly to legacy models coded in standard FORTRAN. The convex relaxations can be denoted as:

(*w*, *z*, *w*^*l*^, *w*^*u*^, *z*^*l*^, *z*^*u*^) ≤ *w *≤ (*w*, *z*, *w*^*l*^, *w*^*u*^, *z*^*l*^, *z*^*u*^)     (11)

where  and  are the convex under-estimates and concave over-estimates of the reformulated problem, respectively.

A linearization strategy [42,44] is then used to generate a Linear Programming (LP) relaxation of the convex NLP created using DAEPACK. The resulting LP is of the form:



where *A*_3 _[*w*^*T *^*z*^*T *^*y*^*T*^]^*T *^≤ *b*_3 _expresses the new linear constraints resulting from the linearization process as illustrated in Figure 1. This linearization technique is ideal because it yields a linear program for which robust solvers exist (e.g. ILOG CPLEX 8.0 [45] and the IBM OSL library [46]). Note that *A*_3_, *b*_3_, *w*^*l *^and *w*^*u *^are updated as *z*^*l *^and *z*^*u *^change in the spatial branch-and-bound algorithm.

### Solution methodology

**Figure 2:**
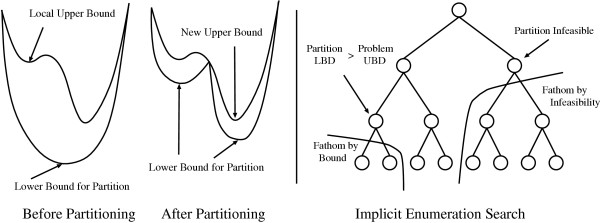
Left: A single branch-and-bound step for a nonconvex function of a continuous variable. Right: Demonstration of implicit enumeration search for a branch-and-bound tree.

The branch-and-bound algorithm [47] can be used as a deterministic method to solve the nonconvex nonlinear problem formulated above. This is illustrated in Figure 2. Branch-and-bound methods depend on generating tight upper and lower bounds for the objective function value at the global solution. A lower bound is generated by solving the convex relaxation of the original nonconvex NLP problem. Any local minimizer for the original NLP problem may serve as an initial upper bound for the objective function value. If the lower bound is sufficiently close to the upper bound, within *ε *tolerance, the algorithm terminates. If not, the feasible region is divided into partitions and lower bounds are generated for the new partitions. Any partition can be removed from further consideration if it is determined that the particular partition cannot contain a better solution than the best solution found so far, or that the lower bounding problem associated with the partition is found to be infeasible. In either case, the partition needs no additional separation. In general, the fathoming criteria can be expressed as follows:

1. If the relaxed problem associated with the partition is infeasible, adding additional constraints will not make the problem feasible. The partition itself is infeasible and hence can be removed from further consideration.

2. If the objective function value of the relaxed problem associated with the current partition is greater or equal to the best solution found so far, then the partition can be removed from further consideration.

Any feasible solution to the original problem may serve as an upper bound for the global solution. The algorithm terminates when the lower bounds for all partitions either exceed or are sufficiently close to the best upper bound. At this point, a global optimum has been determined within the originally preset bounds on the parameter search space. This global optimum is the best value of the objective function. It is noted that multiple points in the parameter space may lead to equivalent values of the objective function.

For the optimization problem shown in Eq. (3), a branch-and-reduce method [48] was implemented. This is an extension of the traditional branch-and-bound method with bound tightening techniques for accelerating the convergence of the algorithm. Within this branch-and-reduce algorithm, infeasible or suboptimal parts of the feasible region are eliminated by using range reduction techniques such as optimality-based and feasibility-based range reduction tests [48-50] or interval analysis techniques [51]. These techniques render tighter variable bounds for a given partition in the search tree, thereby leading to more rapid convergence.

### Didactic example

**Figure 3:**
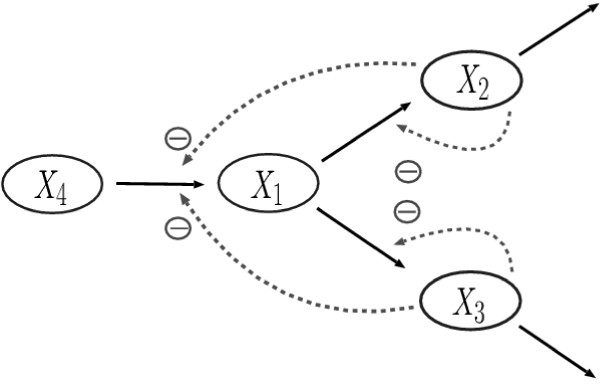
Simple GMA system having several feedback inhibitions.

**Figure 4:**
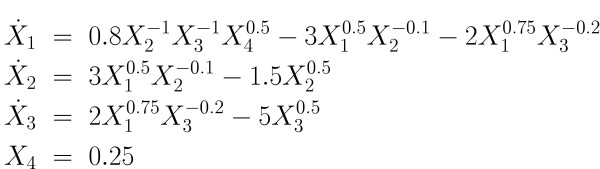
Model equations for simple GMA system.

The identification of model parameters is first illustrated with a simple GMA system adopted from [18] and shown in Figure 3. The system has 3 dependent variables, 1 independent variable and 13 parameters. The initial conditions of the three dependent variables are: *X*_1_(0) = 0.50, *X*_2_(0) = 0.50 and *X*_3_(0) = 1.0. The parameters to be estimated are rate constants and kinetic orders; their true values are shown in Figure 4. It is assumed that the concentration and rate of change for each individual species are known at a series of sampling times.

**Figure 5:**
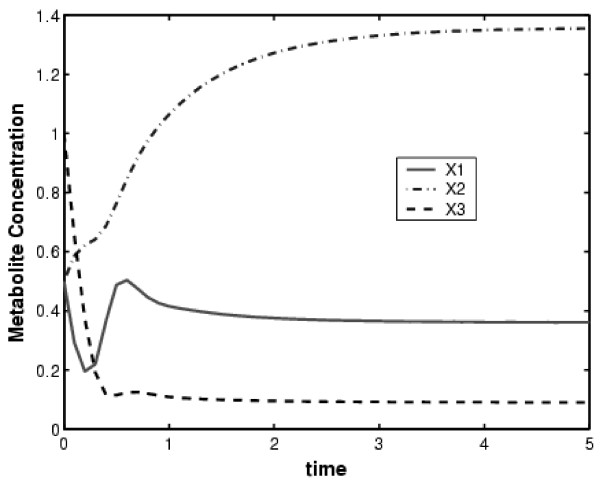
Dynamic response for the simple GMA system having feedback inhibitions.

The chosen initial conditions do not correspond to a steady-state, resulting in a transient response before the system reaches its stable steady-state. Independent variables are held constant and the dynamic data are generated using a single time series. Twelve data points from the transient response are used in this example. This response includes the concentration and rate of change for each individual species. This transient response is shown in Figure 5 and the corresponding data are presented in Table 1. This information is used to formulate the optimization problem given in Eq. (3).

**Table 1:** Time series data of the states and slopes for the didactic example

Data Points	*X*_1_	*X*_2_	*X*_3_			
1	5.00e-1	5.00e-1	1.00	-2.66	1.21e	-3.81
2	2.91e-1	5.86e-1	6.498e-1	-1.52	5.59e-1	-3.17
3	1.96e-1	6.22e-1	3.73e-1	-3.82e-1	2.08e-1	-2.34
4	2.20e-1	6.42e-1	1.90e-1	9.11e-1	2.69e-1	-1.28
5	3.65e-1	6.87e-1	1.16e-1	1.69	6.39e-1	-2.59e-1
6	4.88e-1	7.63e-1	1.15e-1	6.24e-1	8.42e-1	1.08e-1
7	5.04e-1	8.46e-1	1.24e-1	-1.66e-1	7.85e-1	5.54e-2
8	4.75e-1	9.18e-1	1.25e-1	-3.31e-1	6.49e-1	-3.05e-2
9	4.45e-1	9.76e-1	1.197e-1	-2.52e-1	5.25e-1	-6.29e-2
10	4.26e-1	1.02e-1	1.14e-1	-1.43e-1	4.35e-1	-5.59e-2
11	4.15e-1	1.06e-1	1.09e-1	-8.07e-2	3.73e-1	-3.81e-2
12	4.08e-1	1.099e-1	1.06e-1	-5.99e-2	3.26e-1	-2.51e-2

The parameters were estimated by means of the branch-and-reduce global optimization algorithm for various scenarios that differed in both the initial guesses and the bounds for the parameter values. The initial guesses were selected in different ways. In the first series of experiments they were randomly chosen (with uniform distribution) within a predefined range between lower bounds and upper bounds on the parameters. Computational results are presented here with the initial guesses selected as the lower bounds on the parameter search space. Initial parameter guesses can be based on collective experience with GMA and S-systems as described in [18]. Any reasonable initial solution may serve as the initial upper bound on the solution. For our illustration, the upper and lower bounds were selected at 10%, 100%, 200% and 500% around the true value. For instance, in the latter case, they were set at -500% and +500% of the true parameter values, which we knew for this didactic example. The initial bounds on the parameters can thus be computed as:

[*k*_*true *_- 500% × *k*_*true*_*, k*_*true *_+ 500% × *k*_*true*_]     (13)

where *k*_*true *_is the true parameter value. This technique leads to search regions in parameter space centered on the nominal values. However, in the branch-and-reduce algorithm, various range reduction techniques are implemented prior to the global search for the solution. For instance, one may derive tighter variable bounds on a given partition that are not centered around the nominal parameter values. In a realistic situation the true values are of course unknown, but considerable experience has been amassed throughout the years suggesting natural default values. We also included a 0% interval as a check that the true solution was recouped.

The estimated parameter values using both the local and global searches are given in Table 2. The total time required to obtain the global solution, the number of partitions created during the branch-and-reduce procedure and the number of nonconvex and convex problems solved; the objective function values for both local and global solutions are provided in Table 3.

**Table 2:** Estimated parameters using local and global optimization algorithms for the simple GMA system

Actual Parameters	Estimated parameters with varying bounds									
	
	0 %		10 %		100 %		200 %		500 %	
	
	Local	Global	Local	Global	Local	Global	Local	Global	Local	Global
0.8	0.8	0.8	0.8	0.8	0.799	0.799	-0.8	0.8	0.8	0.8
1.0	1.0	1.0	1.0	1.0	1.0	1.0	3.0	1.0	1.0	1.0
1.0	1.0	1.0	1.0	1.0	1.0	1.0	-1.0	1.0	1.0	1.0
3.0	3.0	3.0	3.0	3.0	2.99	2.99	-1.09	3.0	3.0	3.0
0.5	0.5	0.5	0.5	0.5	0.5	0.5	-0.45	0.5	0.5	0.5
0.1	0.1	0.1	0.1	0.1	0.1	0.1	0.3	0.1	0.1	0.1
2.0	2.0	2.0	2.0	2.0	2.0	2.0	1.019	2.0	2.0	1.999
0.75	0.75	0.75	0.75	0.75	0.75	0.75	-0.40	0.75	0.75	0.749
0.2	0.2	0.2	0.2	0.2	0.199	0.199	-0.2	0.2	0.2	0.2
1.5	1.5	1.5	1.5	1.5	1.499	1.499	-1.5	1.5	1.5	1.5
0.5	0.5	0.5	0.5	0.5	0.499	0.499	-0.5	0.499	0.5	0.499
5.0	5.0	5.0	5.0	5.0	5.0	5.0	6.074	5.0	5.0	4.999
0.5	0.5	0.5	0.5	0.5	0.499	0.499	0.611	0.499	0.5	0.5

**Table 3:** Total time taken for global solution, number of partitions created during the Branch-and-Reduce algorithm, nonconvex and convex problems solved, and objective function values for both local and global solution methods for the small GMA system.

	Time for Global Sol (sec)	No of Partitions	Nonconvex Problems	Convex Problems	Obj fun Value (Global)	Obj fun Value (Local)
0 %	0.252	1	1	1	0.0	0.0
10 %	0.662	1	1	1	0.0	0.0
100 %	1.453	1	1	1	0.0	0.0
200 %	12.005	11	13	11	0.0	0.917
300 %	14.412	13	15	13	0.0	0.884
400 %	10.372	9	11	9	0.0	0.867
500 %	20.042	15	17	15	0.0	0.846

The numerical results demonstrate that both the local and global solvers give the same solution when the parameter space is small and the bounds are tight. When the bounds are increased beyond 100%, the local solver may fail to find the true parameters, while the global solver still succeeds. For instance, one notes that the local search with 200% bounds yielded a different solution, which however was inferior, as indicated by the value of the objective function.

### Case study

**Figure 6:**
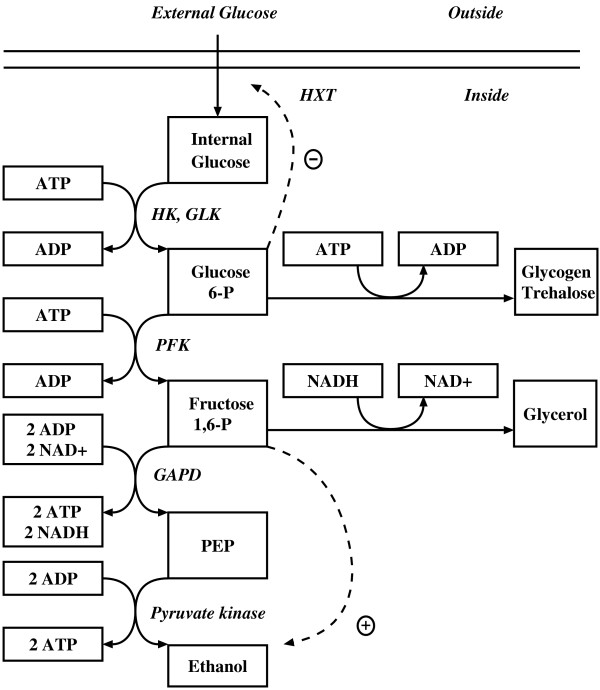
Simplified model of anaerobic fermentation of glucose to ethanol, glycerol, and polysaccharides in Saccharomyces Cerevisiae.

**Figure 7:**
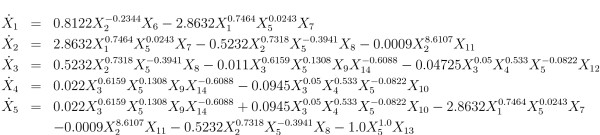
GMA model equations for fermentation model in Figure 6.

As a more complex illustration, consider the fermentation pathway in *Saccharomyces cerevisiae *described in [26]. This is a relatively simple metabolic pathway system of which the structural and numerical specifications are however directly based on careful kinetic experiments and biochemical analyses [52]. The metabolic pathway map is given in Figure 6. The GMA model equations adapted from [26,53] are given in Figure 7. The model has 5 dependent variables, 9 independent variables and 19 unknown rate constant and kinetic order parameters.

According to Galazzo and Bailey [52] and Curto et al. [26], the observed concentrations (mM) of the dependent variables at steady state are: *X*_1_(G_*In*_) – Internal Glucose = 0.0346, *X*_2_(G6P) – Glucose-6-phosphate = 1.011, *X*_3_(FDP) – Fructose-1,6-diphosphate = 9.1876, *X*_4_(PEP) – Phosphoenolpyruvate = 0.0095, and *X*_5 _– Adenosine triphosphate (ATP) = 1.1278. The values of the independent variables (*mM min*^-1^) are: *X*_6 _– Glucose uptake = 19.7, *X*_7 _– Hexokinase = 68.5, *X*_8 _– Phosphofructokinase = 31.7, *X*_9 _– Glyceraldehyde-3-phosphate dehydrogenase = 49.9, *X*_10 _– Pyruvate kinase = 3,440, *X*_11 _– Polysaccharide production (glycogen + trehalose) = 14.31, *X*_12 _– Glycerol production = 203, *X*_13 _– ATPase = 25.1, and *X*_14 _– *NAD*^+^*/NADH *ratio = 0.042.

**Figure 8:**
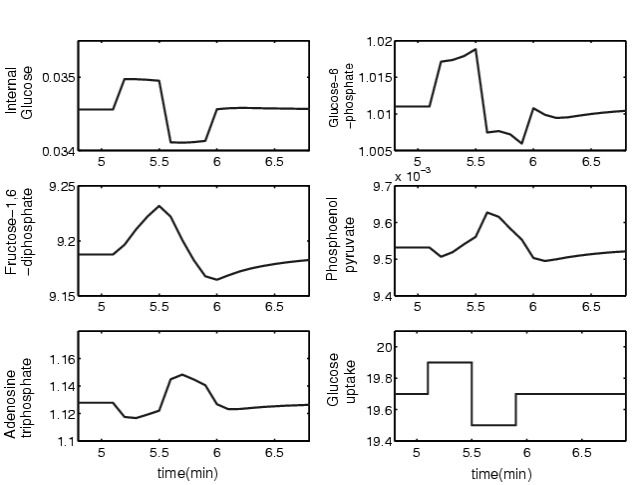
Dynamic response of independent variables when the external glucose uptake is subjected to step changes.

A typical dynamic response for the system is shown in Figure 8. It was obtained by subjecting the glucose uptake to artificial step changes, which could have been implemented experimentally through externally controlled changes in substrate availability. Illustrative data were obtained by integrating the system and collecting the state and the slope values for each dependent variable every 6 seconds over a time horizon of length *P*. For a single time series, the horizon length is the number of data points collected for the state and slope values that are used in the optimization formulation.

For any given scenario, *nP *equations were written, and these served as the nonlinear equality constraints in the formulation given in Equation 3, which includes the unknown parameters (*θ*) that were to be estimated. For a single time series, 10 data samples for the states and slope values were collected right after the first step change was introduced. The transient response data are presented in Table 4. The resulting optimization formulation thus required 70 variables (parameters) and 50 nonconvex constraints. The convex relaxation resulted in 276 total variables and 832 total convex constraints.

**Table 4:** Time series data of the states and slopes for the anaerobic fermentation case study (In every cell, the first value represents the state and the second value represents the slope)

Data Points	X_6 _ External Glucose	*X*_1 _ (G_In_)	*X*_2 _ (G6P)	*X*_3 _ (FDP)	*X*_4 _ (PEP)	*X*_5 _ (ATP)
1	19.7	3.46e-2	1.011	9.188	9.53e-3	1.1278
		-1.78e-15	1.10e-13	-1.97e-9	3.93e-9	3.93e-9
2	19.9	3.46e-2	1.011	9.188	9.53e-3	1.1278
		1.62e-1	1.10e-13	-19.7e-9	3.93e-9	3.93e-9
3	19.9	3.498e-2	1.017	9.196	9.51e-3	1.1175
		-7.399e-5	1.021e-2	1.38e-1	-9.79e-6	-4.68e-2
4	19.9	3.498e-2	1.017	9.21	9.52e-3	1.1167
		-3.69e-5	2.04e-3	1.32e-1	2.04e-4	1.76e-2
5	19.9	3.497e-2	1.018	9.22	9.54e-3	1.1193
		-1.177e-4	8.28e-3	1.06e-1	2.16e-4	2.91e-2
6	19.5	3.495e-2	1.019	9.232	9.56e-3	1.1221
		-3.24e-1	9.67e-3	8.59e-2	1.821e-4	2.568e-2
7	19.5	3.41e-2	1.0075	9.22	9.63e-3	1.1449
		5.68e-5	-1.36e-2	-2.03e-1	1.595e-4	1.131e-1
8	19.5	3.41e-2	1.008	9.201	9.616e-3	1.148
		-1.34e-5	2.35e-3	-2.03e-1	-2.85e-4	-1.79e-2
9	19.5	3.412	1.0072	9.182	9.58e-3	1.145
		1.47e-4	-1.02e-2	-1.63e-1	-3.26e-4	-4.32e-2
10	19.7	3.41e-2	1.006	9.168	9.55e-3	1.1406
		1.62e-1	-1.38e-2	-1.32e-1	-2.77e-4	-3.91e-2

The parameters were estimated with the MINOS nonconvex NLP solver for local searches and the branch-and-reduce algorithm for the global searches. As in the didactic example, we explored various scenarios that again differed with respect to the initial guesses for the parameters as well as the bounds on the parameter space to be searched. The lower and upper bounds on the parameters were selected at 100%, 200%, 300 %, and 500% around the true value. The initial guesses for the parameters were selected to be the lower bounds of the parameters search space.

It is known that the performance of any MINOS NLP solver is significantly affected by the specified tolerances. The convergence tolerances such as row, optimality and feasibility tolerances were set to 10^-5^. The maximum number of iterations was set to 5000, and the maximum number of major and minor iterations between successive linearizations of nonlinear constraints was set to a value of 60. During the branch-and-reduce algorithm, only the parameters (*γ *and *p*) were considered for branching. The ratios of the difference in the current bounds and the difference in the original bounds for all variables (parameters) were computed. The particular variable with the worst (largest) ratio was then selected for branching. The algorithm was implemented using best-first search strategy and was able to guarantee the global solution within an *ε *= 0.0001 tolerance.

The computational results were generated using MINOS for NLP solution and CPLEX for the LP solution. An Athlon 1900+ dual processor machine was used with Linux Debian OS. The computational results demonstrate that the chosen optimization technique guarantees convergence to the global solution for all Δ values tested. The global solution time, number of partitions, nonconvex and convex problems solved during the branch-and-reduce search, and the objective function value for both the local and global solutions, are given in Table 5. The estimated parameters for the different scenarios are shown in Table 6.

**Table 5:** Estimated parameters for anaerobic fermentation pathway using Branch-and-Reduce algorithm

Actual Parameters (θ)	Estimated parameters with varying bounds					
	
	100 %		300 %		500 %	
	
	Local	Global	Local	Global	Local	Global
0.8122	0.8112	0.8122	0.8112	0.8122	0.8112	0.8122
0.2344	0.2393	0.2344	0.2396	0.2344	0.2398	0.2344
2.8632	2.8557	2.8632	2.8521	2.8632	2.8485	2.8632
0.7464	0.7460	0.7464	0.7456	0.7464	0.7452	0.7464
0.0243	0.0259	0.0243	0.0256	0.0243	0.0253	0.0243
0.5232	0.5221	0.5232	0.5214	0.5232	0.5206	0.5232
0.7318	0.7364	0.7318	0.7373	0.7318	0.7382	0.7318
0.3941	0.3941	0.3941	0.3949	0.3941	0.3954	0.3941
0.0009	0.0018	0.0009	0.0036	0.0009	0.0054	0.0009
8.6107	0.0000	8.6107	0.0000	8.6107	0.0000	8.6107
0.011	0.0109	0.011	0.0109	0.011	0.0109	0.011
0.6159	0.6169	0.6159	0.6177	0.6159	0.6185	0.6159
0.1308	0.1302	0.1308	0.1305	0.1308	0.1308	0.1308
0.04725	0.0337	0.034	0.0174	0.0467	0.0089	0.0464
0.05	0.1	0.1	0.2	0.0517	0.3000	0.0528
0.533	0.4852	0.486	0.3915	0.531	0.2977	0.5303
0.0822	0.0638	0.063	0.026	0.0816	-0.011	0.0811
1.0	0.9975	1.0000	0.9937	1.0000	0.9899	1.0000
1.0	0.9979	1.0000	1.0013	1.0000	1.0047	1.0000

**Table 6:** Result of estimation analysis with the anaerobic fermentation pathway model. Total time required for global solution, number of partitions created during the Branch-and-Reduce search, number of nonconvex and convex problems solved, and the value of the objective function for both local and global solution methods.

	Time for Global Sol (sec)	No of Partitions	Nonconvex Problems	Convex Problems	Obj fun Value (Global)	Obj fun Value (Local)
100 %	38.13	27	15	27	0.0	1.36 × 10^-4^
200 %	19.28	9	6	9	0.0	1.34 × 10^-4^
300 %	56.98	27	15	27	1.61 × 10^-7^	1.31 × 10^-4^
400 %	58.39	25	14	25	0.0	1.31 × 10^-4^
500 %	57.15	25	14	25	3.51 × 10^-8^	1.25 × 10^-4^

## Conclusion

The first step of any modeling effort is the definition of symbolic equations and the numerical definition of their parameter values. In the past, the latter has typically been accomplished by reformulating literature information on local processes, such as enzyme-catalyzed reaction steps, within the biological system of interest. High-throughput data are beginning to change this situation dramatically. Instead of working from the bottom up, it is becoming possible to infer the structure and regulation of biological systems from measured time profiles. An important component of this inference is the efficient estimation of parameters, which in the case of GMA and S-systems within BST can directly be interpreted as biological features, thereby potentially yielding novel insights.

We have shown here that the parameter estimation task may be posed as a nonconvex nonlinear optimization problem. Consequently, deterministic global optimization techniques can be applied, and among them the branch-and-reduce algorithm appears to be a very suitable choice, because it is guaranteed to find the global solution. This is in stark contrast to local solvers, such as nonlinear regression algorithms, which may not be able to converge to the global solution when the parameter search space is large or the error surface is ragged. As a proof of concept, we demonstrated the features of the global search method with a didactic example and a simple yet realistic biological case study, which has been used by others for the exploration of new methods [18,26-29]. In order not to cloud the demonstration of the functioning of the branch-and-reduce search algorithm, both examples were selected free of experimental error. While this may seem somewhat artificial, the omission of noise was intentional (a) in order to see how the method performs under ideal conditions and (b) because a preparatory smoothing step can significantly reduce if not eliminate noise. Nonetheless, it is now necessary to consider data that are corrupted by noise, and to compare the efficacy of the branch-and-reduce method with alternate methods such as nonlinear regression and genetic algorithms.

For the limited range of illustrative examples shown here, the computational results demonstrate that the branch-and-reduce algorithm is fast and reliable. Of course, it is difficult to judge in general to what degree this algorithm, combined with estimation of species concentration rate of change, outperforms other methods, but it seems clear that direct parameter estimation in systems of differential equations can be extremely time consuming. As a case in point, Kikuchi *et al*. [4] used a genetic algorithm to identify the parameters of a five-variable S-system from noise-free data and showed that every cycle of the genetic algorithm took about 10 hours on a cluster of over 1,000 CPUs and that seven cycles were needed to complete the identification. Voit and Almeida [9] demonstrated that this computation time may be reduced drastically if the estimation of slopes, as we used here, is employed to replace the differential equations with sets of nonlinear algebraic equations. Still they found the subsequent regression to be slow and not necessarily reliable, because of convergence to local minima or no convergence at all. Irrespective of computational speed, the branch-and-reduce algorithm employed here was shown to be successful in rather quickly finding global solutions within an *ε *tolerance, which is a great advance, because local search methods cannot guarantee convergence to the true solution.
